# Cardiovascular health knowledge and preventive practices in people living with HIV in Kenya

**DOI:** 10.1186/s12879-015-1157-8

**Published:** 2015-10-14

**Authors:** Tecla M. Temu, Nicholas Kirui, Celestine Wanjalla, Alfred M. Ndungu, Jemima H. Kamano, Thomas S. Inui, Gerald S. Bloomfield

**Affiliations:** 1 Department of Medicine, School of Medicine, College of Health Sciences, Moi University, Eldoret, Kenya; 2 Department of Epidemiology, Brown University School of Public Health, Providence, RI USA; 3 AMPATH Partnership, Eldoret, Kenya; 4 Division of Medicine, Moi Teaching and Referral Hospital, Eldoret, Kenya; 5 Department of Medicine, Vanderbilt University, Nashville, TN USA; 6 Department of Statistics, North Dakota State University, Fargo, ND USA; 7 Department of Medicine, Indiana University, Indianapolis, USA; 8 Department of Medicine, Duke Clinical Research Institute and Duke Global Health Institute, Duke University, Durham, NC USA

**Keywords:** HIV, Cardiovascular diseases, Sub Saharan Africa, Kenya, Knowledge, Perception

## Abstract

**Background:**

Traditional cardiovascular disease (CVD) risk factors contribute to increase risk of CVD in people living with HIV (PLWH). Of all world regions, sub-Saharan Africa has the highest prevalence of HIV yet little is known about PLWH’s CVD knowledge and self- perceived risk for coronary heart disease (CHD). In this study, we assessed PLWH’s knowledge, perception and attitude towards cardiovascular diseases and their prevention.

**Methods:**

We conducted a cross-sectional study in the largest HIV care program in western Kenya. Trained research assistants used validated questionnaires to assess CVD risk patterns. We used logistic regression analysis to identify associations between knowledge with demographic variables, HIV disease characteristics, and individuals CVD risk patterns.

**Results:**

There were 300 participants in the study; median age (IQR) was 40 (33–46) years and 64 % women. The prevalence of dyslipidemia, overweight and obesity were 70 %, 33 % and 8 %, respectively. Participant’s knowledge of risk factors was low with a mean (SD) score of 1.3 (1.3) out of possible 10. Most (77.7 %) could not identify any warning signs for heart attack. Higher education was a strong predictor of CVD risk knowledge (6.72, 95 % CI 1.98-22.84, *P* < 0.0001). Self-risk perception towards CHD was low (31 %) and majority had inappropriate attitude towards CVD risk reduction.

**Conclusion:**

Despite a high burden of cardiovascular risk factors, PLWH in Kenya lack CVD knowledge and do not perceived themselves at risk for CHD. These results emphasis the need for behavior changes interventions to address the stigma and promote positive health behaviors among the high risk HIV population in Kenya.

**Electronic supplementary material:**

The online version of this article (doi:10.1186/s12879-015-1157-8) contains supplementary material, which is available to authorized users.

## Background

Over 22.5 million people living with human immunodeficiency virus (PLWH) reside in sub-Sahara Africa (SSA), representing 68 % of the global HIV burden [1]. At the same time, CVD accounts for 11 % of deaths in SSA and the number of deaths due to CVD have increased by 81 % between 1990 and 2013 [2, 3]. The burden of CVD is expected to double by 2020 [3]. Half of CVD deaths in SSA occur among people aged between 30 and 69, which is 10 years earlier than in North America and Europe [http://www.ichealth.org]. Mortality and morbidity in this age group has major social and economic consequences depriving families of parents, work places of employees and communities of leaders.

There is a greater than expected risk of CVD in PLWH [4, 5]. The commonly reported CVD manifestations include carotid atherosclerosis, large vessel vascular disease, and coronary artery disease [6]. Increased CVD risk in PLWH is attributed to chronic inflammation, immune activation associated with HIV-infection, opportunistic infections and traditional CVD risk factors including dyslipidemia associated with antiretroviral therapy (ART) [6]. Although the overall risks and benefit of ART for reducing cardiovascular events is still controversial, continuous ART has been associated with better CVD outcomes than the ART interruption as demonstrated by the Strategies for Management of Antiretroviral Therapy (SMART) study [7]. Therefore, among the many factors, modifiable risk factors such hypertension, obesity, dyslipidemia, smoking and lack of physical activity seem to play a central role in accelerating the risk of CVD in this population [8]. Acute myocardial infarction risk increases exponentially with each additional cardiovascular risk factor in PLWH compared to having no CVD risk factors [8]. Most studies reporting high CVD risk in PLWH have been performed in developed countries [9, 10]. The few data from SSA also suggest greater CVD risk in PLWH [11–14].

Knowledge of CVD risk factors is a critical prerequisite for an individual to make behavioral changes leading towards optimal cardiovascular health [15, 16]. Patients with good knowledge of heart attack warning signs are more likely to present for treatment earlier with better outcomes [17]. Individuals with a greater knowledge of coronary heart disease (CHD) risk factors also have higher perceived risk [18]. According to health behavior model, individuals with high perceived risk for CVD are more likely to adopt healthy behaviors such as weight loss and smoking cessation [16]. Cultural biases and socio- economics are of great significance in resource-limited areas such as SSA and these factors are lacking in many studies in this field [19]. Therefore, measuring knowledge level of PLWH on CVD is a crucial step in the designing of future prevention strategies for PLWH in SSA. In this study, we examined PLWH’s CVD risk factor profile, knowledge of CVD risk factors and warning sign of heart attack, risk perception and currents practices towards cardiovascular health in Kenya, a country with an overall 6 % prevalence of HIV and rising CVD burden [20]. We are not aware of any studies that have examined the knowledge of CVD in PLWH in SSA. Results from this study will be used to guide public health interventions to reduce cardiovascular disease risk in PLWH in SSA.

## Methods

### Ethical consideration

The Institutional Research and Ethics Committee of Moi University School of Medicine approved the study. All participants provided a written informed consent. Any findings that were felt to warrant immediate medical attention were reported to the participant and their physician.

### Study site

Participants were recruited from the outpatient HIV clinic of Moi Teaching and Referral Hospital (MTRH) in Eldoret within the Academic Model Providing Access to Healthcare (AMPATH) program which provides care to >150,000 adults and children living with HIV/AIDS throughout Western Kenya [21]. The AMPATH program is a collaboration between MTRH, Moi University School of Medicine, and a consortium of North American universities that focuses on improving the health of the people of Western Kenya as previously described [22]. This site was chosen because of its broad mix of patients including a mix of urban middle class, urban poor and the rural population. Moi University is home to an NHLBI-sponsored Cardiovascular and Pulmonary Disease Center of Excellence in Cardiovascular and Pulmonary Diseases (COE) [23], and is the hub of clinical research in cardiopulmonary diseases in Western Kenya [24]. HIV-infected patients fulfilling Kenyan national criteria for ART are started on treatment and are seen monthly at the AMPATH clinic. Criteria for starting ART at the time of the study included all adult PLWH with CD4 T cell count <350 cells/mm^3^ irrespective of WHO stage and stage III/ IV disease regardless of CD4 T cell count. The first-line ART regimen consisted of either tenofovir/ lamivudine or zidovudine/lamivudine + nevirapine or efavirenz. Protease inhibitors (PIs) were only given as second-line ART in accordance with Kenya national guidelines for antiretroviral drug therapy [25].

### Study population and design

As part of a larger study to assess the prevalence of cardiovascular risk factors in PLWH, we conducted a descriptive cross-sectional study to investigate knowledge, attitude and self-risk perception towards CVD in a sample of PLWH in Western Kenya attending AMPATH HIV clinic. The study sample was a convenience sample of patients consisting of two groups of adults (aged ≥18 years old) with a diagnosis of HIV; adult PLWH not yet on ART, and adult PLWH on ART regardless of the length of time they have been on ART. Participants were required to be able give a written informed consent and respond to questions. Exclusion criteria included any patients with recorded history of CVD. Consecutive adult PLWH who met the criteria were asked to participate.

### Data collection

Between July to September 2014, research assistants invited patients who presented for their routine clinic appointment to participate in the study. Data were collected by structured questionnaires, physical examination and venous blood sample analysis. A trained research assistant administered the questionnaire in English, Swahili or a local language (Additional file 1). Each interview lasted approximately 30 min followed by physical measurements. Participants were asked return to clinic the following day after fasting for eight hours for biochemical assessments. The research participants were expected to complete all the components of the research examination on the second visit whenever possible. Participants received compensation to cover for transportation.

### Questionnaire

Items in the questionnaire were constructed from multiple validated surveys [26–28]. The draft questionnaire was piloted with 25 people to measure its validity prior to administering to the study population. The final questionnaire contained three sections: social and demographic characteristics, knowledge of CVD and CHD; and attitude and perception towards CHD and CHD prevention (Additional file 1). Open ended and prompt questions were incorporated in all three sections.

### Social demographic characteristics and medical history

Included information on age, sex, marital status, education level, medical history including HIV duration and family history of CVD. Cardiovascular risk factors assessment was done using items from the World Health Organization non -communicable disease STEPwise approach to CVD risk factor surveillance [29]. Information on use of ART, the type of ART regime used and latest CD4+ lymphocyte count was obtained from the participant’s medical records.

### Knowledge of CVD and CHD

Participants’ were asked to list up to ten CVD risks factors and seven CHD warning signs. Responses were graded as correct or incorrect using guidelines from the American Heart Association as the gold standard [30]. Acceptable responses included age, high blood pressure (BP), high cholesterol, smoking, obesity, family history, diabetes, stress, alcohol use, and physical inactivity. Acceptable warning symptoms of heart attack included dizziness, shortness of breath, sweating, loss of consciousness, chest pain, nausea/vomiting, and pain in the teeth, arm, or jaw. Each response was scored 0 for a wrong answer or 1 for the correct answer, with a minimum score of 0 and a maximum score of 10 points or 7 points for CVD risk factors and CHD warning signs, respectively. Participants were also asked to identify action they would take in case they suspected a heart attack.

### Attitude and Perception towards CVD and CHD prevention

This section contained questions to assess participants’ self-perceived risk for heart diseases, perceived seriousness of CHD, attitude and beliefs, and current practices for CHD prevention. In addition, participant’s ways of accessing health information was also recorded.

### Physical measurements

Height, weight, waist, and hip circumference were measured. Body mass index (BMI) was calculated using the measured weight and height (kg/m^2^). Blood pressure was measured using an automated digital sphygmomanometer (Omron Hem 712c, Omron Healthcare, Kyoto, Japan). Bilateral BP was taken twice, with two-minute interval between measurements. High BP was defined as systolic BP ≥ 140 mm Hg, diastolic BP ≥ 90 mm Hg, or currently on antihypertensive drug treatment. Central obesity was defined as waist circumference of ≥80 cm (women) and ≥90 cm (men).

### Biochemical assessments

Blood samples from individuals fasting for >8 h were collected by venipuncture in BD vacutainer tubes. Samples were typically processed within 4 h of collection. A five-milliliter BD vacutainer tube was centrifuged at 3000 rpm for 4 min and serum was collected. Biochemical analysis was performed using the COBAS Integra 400 plus chemistry analyzer to determine blood glucose (FBG) and lipid profiles from the serum sample. Diabetes mellitus was defined as FBG ≥ 126 mg/dl and dyslipidemia was defined as total cholesterol (TC) ≥200 mg/dl or HDL-C <40 mg/dl, LDL-C ≥130 mg/dl, and triglycerides >350 mg/dl by the American Heart Association and American College of Cardiology Foundation [31].

### Statistical analysis

The analysis was completed using SAS® University Edition. Descriptive data are presented as means and percentage. Continuous data are presented as median and interquartile range (IQR). Chi Square tests were performed to investigate the association of knowledge level with categorical variables. Logistic regression analyses were done to identify univariate associations between the ability to identify risk factors for heart attack and warning symptoms. A multivariate logistic regression model was fit to analyze these associations after controlling for demographic variables, HIV disease characteristics, and individuals CVD risk patterns. Chi-square test was performed to measure association between heart disease risk perception and CVD risk status. CVD risk status was defined as a binary outcome as having one or more of the established risk factors for CVD. Statistical significance was set at *P* < 0.05.

## Results

### Demographic and health characteristics

Of 300 participants, 192 (64 %) were female and the median (IQR) age was 39. 7(33.6 – 46.3) years. Over half (55 %) were on ART, 38.6 % had more that 8 years of formal education. The median (IQR) HIV and ART use duration were 4.6 (1.7 – 7.9) and 4.8 (2.7– 7.8) years, respectively. Additional demographic characteristics are reported in Table 1.

**Table 1:** Characteristics of the study participants

Characteristics	Total (*n* = 300)
Median age, y (IQR)	39.7 (33.6 – 46.3)
Female, n (%)	192 (64)
Overall age data (y)	
18–39	146 (49)
40–59	142 (47)
60–80	12 (4)
Education, n (%)	
Tertiary (>12)	40 (13.3)
Secondary (9 – 12 grade)	76 (25.3)
Primary (0–8 grade)	131 (43.7)
Never attended school	53 (17.7)
Marital status, n (%)	
Single	45 (15)
Married	235 (78.3)
Widowed	16 (5.4)
Separated	4 (1.3)
Current professional status, n (%)	
Employed	74 (25)
Self-employed	132 (44)
Unemployed	94 (31)
Family History, n (%)	
High Cholesterol	2 (0.7)
Diabetes	9 (3)
High blood pressure	4 (1.3)
Myocardial infraction	1 (0.3)
HIV related characteristics	
WHO HIV stage, n (%)	
Stage 1&2	204 (68)
Stage 3&4	96 (32)
Median HIV infection duration (y), IQR	4.6 (1.7–7.9)
Median ART duration (y), IQR	4.8 (2.7– 7.8)
Current CD^4^+ T cell, (cells/mm^3^), SD	413 (215)
Protease inhibitor use	11 (3.7)
ART-naive	136 (45)

*ART* antiretroviral therapy

**Fig. 1:**
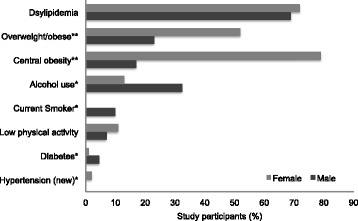
Prevalence of selected CVD risk factors in the study population. All (*n* = 300) participants were included. Current smokers are those who have responded “yes” to smoking. Alcohol use was defined by intake of at least one drink within the previous month. Central obesity was defined as waist circumference of ≥80 cm (women) and ≥90 cm (men). BMI (kg/m^2^) definition are; overweight 25.0-29.9 and obesity ≥ 30.0. ^*^ *P* <0.05, ^**^ *P* < 0.001

Figure 1 shows the distribution of CVD risk factors among participants. The prevalence of dyslipidemia, central obesity, and overweight/obese was 70 %, 57 %, and 41 % respectively. Average (SD) BMI was 24.4 (5.6). Mean (SD) total cholesterol, LDL cholesterol and HDL was 174 mg/dl (3.8), 104 mg/dl [18] and 50 mg/dl [22] respectively. Alcohol and tobacco use were reported in 20 % and 3.3 % of the participants, respectively. Most participants (92 %) reported being physically active and less than 3 % were found to have high BP or diabetes. Mean (SD) fasting glucose for our participants was 90 mg/dl [17]. None of our participants reported receiving treatment for dyslipidemia, hypertension or diabetes. Almost 44 % of the participants had two or more risk factors. Women were likely to have overweight/obesity (*P* = 0.03), to have raised BP (*P* < 0.0001), central obesity (*P* = 0.001), and less likely to report tobacco use (*P* < 0.0001), alcohol use (*P* = 0.001) than men.

### Knowledge of CVD risk factors

**Fig. 2:**
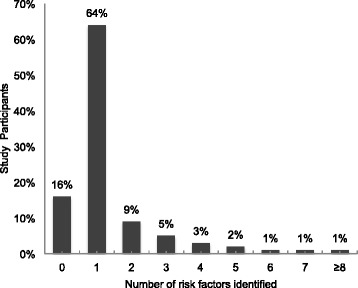
CVD risk factors as identified by participants

In an open-ended question, participants were asked to list risk factors for CVD. Table 2 shows unprompted responses for the perceived risk factors of CVD. Knowledge of CVD risk factor was assessed as a continuous variable. The mean (SD) score for CVD risk factors identified by participants was 1.3 (1.3) out of possible 10. The most common risk factor identified by the group was stress (74 %) followed by physical inactivity (13 %) and obesity/overweight (9 %). Less than 10 % of participants correctly identified an established biological risk factor for CVD while sixteen percent of participants could not identify any risk factor at all (Fig. 2).

**Table 2:** CVD risk factors as identified by participants

Risk factors	N (%)
Don’t know	47 (16)
Don’t know	47 (16)
Stress	221 (73.7)
Physical inactivity	38 (12.6)
Overweight/obese	28 (9.3)
Raised blood pressure	27 (9.0)
Alcohol overuse	23 (7.6)
Diabetes	15 (5.0)
High Cholesterol	15 (5.0)
Smoking/Tobacco use	12 (4.0)
Age	7 (2.3)
Family history	4 (1.3)

In univariate analysis, factors significantly associated with ability to identify ≥ 1 CVD and CVD risk factor were >12 years of education (OR 4.18, 95 % CI 1.74-10.07, *P* < 0.0001) and family history of diabetes (OR 5.21, 95 % CI 0.99-27.37, *P* = 0.05). However in the multivariable analysis, only >12 years of education was independently associated with the ability to identify ≥ 1 CVD risk factor (OR 6.72, 95 % CI 1.98-22.84, *P* < 0.000). No significant association was found between risk knowledge and risk status (*P* = 0.26).

### *Knowledge of heart attack* symptoms

**Fig. 3:**
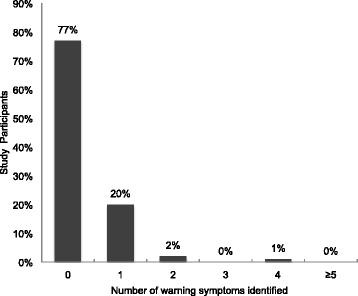
Heart attack warning signs as identified by participants

Participants were also asked to list heart attack warning signs. Table 3 shows participant knowledge of heart attack symptoms. The mean (SD) score for heart attack symptoms identified by the group was 0.28 (0.6) out of 7. With the exception of loss of consciousness and difficulty breathing identified by 10 % of participants, a very small proportion (<3 %) could identify the other established warning signs. Over three quarters of participants (77 %) could not identify one warning sign (Fig. 3). No significant associations were observed between heart attack symptoms knowledge (defined as identification of ≥ 1 heart attack warning signs) and the demographic variables. An overwhelming majority reported that if they thought they were having a heart attack they will go to a health care facility (95 %) while the remaining five percent noted they will go to the pharmacy or use traditional medicines.

**Table 3:** Heart attack warning signs as identified by participants

Warning signs	N (%)
Don’t know	233 (77.3)
Loss of consciousness	31 (10.3)
Dyspnea	30 (10.0)
Dizziness or light headedness	10 (3.3)
Chest pain	5 (1.7)
Excessive sweating	3 (1.0)
Nausea/Vomiting	2 (0.7)
Pain in the teeth, jaw or arm	2 (0.7)

### Perceived seriousness of heart diseases

Participants were asked in an open-ended question to list the greatest health problem in PLWH. Most (53 %) noted stress, AIDS (17 %) and depression (14 %). Heart diseases were noted by 1 % of the participants, all male (*P* = 0.03). When asked to list the leading cause of death in PLWH, opportunistic infections such as tuberculosis were noted by 94 % of participants.

### Self-Perceived risk for heart disease

Only one third of the participants agreed with the statement “I am at high risk for heart disease”. Older women were more likely to agree with this statement (*P* = 0.04) than men. Less than half of the participants agreed that CHD is preventable and that changing lifestyle behavior would reduce their chances of developing CHD. No association was found between risk status and self perceived risk to CHD (*P* = 0.1937). Other beliefs, or and attitude about heart diseases risk and prevention are shown on Table 4.

**Table 4:** Attitude/beliefs towards CHD risk and current practices towards CHD prevention

Attitudes towards CHD prevention	Number	Percent
Changing lifestyle behavior will cut down my chances of developing heart diseases *(HBM Self efficacy)*	131	(41)
I am at risk for heart disease. *(HBM perceived risk)*	208	(31)
Heart disease is preventable	37	(12)
My present weight is too high for my health	29	(10)
I need to cut down on the amount of food I eat	27	(9)
Current practices towards cardiovascular health		
In the past year, have you		
Had your blood pressure measured	300	(100)
Tried to cut down weight	11	(4)
Increase physical activity/exercise	9	(3)
Had cholesterol or blood sugar checked	8	(3)
Attempted to cut down on unhealthy foods	7	(2)
Reduce/quit smoking	1	(1)

Questions on attitude towards CHD prevention were based on a 5-scale likert questions (strongly disagree, disagree, I do not know, agree and strongly agree. We classified five options into three categories as ‘agree, ‘disagree’ and ‘I do not know’. Only ‘strongly agree’ and ‘agree’ answers were merged and presented in the table

Preventive practices against CHD were also assessed (Table 4). Less than 4 % had ever had their blood sugar or cholesterol monitored. In addition, <5 % of participants reported recreational exercise, cutting down on unhealthy foods, or smoking cessation.

### Source of heart disease information

Over half of our participants reported hearing about CHD in the preceding year. Main source of CVD information were television (51 %), radio (44 %), magazine (19 %) or internet (4 %) rather than a health care professional (4 %). Only 3 % reported to have discussed CVD with a health care provider.

## Discussion

Despite the high frequency of CVD risk factors among participants, we found that PLWH in western Kenya had poor knowledge of both CVD risk factors and heart attack warning signs and had low self-perceived risk of CHD. Preventive practices towards health heart were also lacking. Few participants cited heath care professionals as source of CHD information. Our study suggests that CVD knowledge, a critical ingredient in understanding risk, is low. The information obtained from this study can inform future strategies and interventions for CVD prevention in PLWH.

Despite participants being relatively young, the prevalence of multiple CVD risk factors was high. Obesity and dyslipidemia was diagnosed in over half of our patients. One third of our participants had more than one CVD risk factor. Protease inhibitors (PI) have been associated with increased risk of myocardial infarction [32]. It is unlikely that protease inhibitors were the major drivers of obesity and dyslipidemia because less than 4 % of the participants had been on a PI-based regimen. Perhaps other factors, including but not limited to traditional risk factors, chronic inflammation associated with HIV and non-PI based ART regiments may also be responsible. The link between traditional risk factors and atherosclerosis has been well documented in the developed countries [10]. But a recent study in South Africa by *Schoffelen* et al. demonstrated, for the first time, that subclinical atherosclerosis was strongly associated with traditional risk factors rather than the HIV-related factors in PLWH supporting earlier findings from the developed countries [8, 10, 14]. This report together with our data emphasize the need to integrate CVD risk assessment and implementing cost effective strategies to manage CVD risk factors into the current HIV care in resource-limited countries.

Knowledge of the established CVD risk factors was very low. This is in accordance with a study conducted in PLWH in the US [33]. The most common risk factor identified in the present study was stress, consistent with findings the general population from non-Western countries [34, 35]. Established risk factors, including those highly prevalent in our HIV population such as high cholesterol levels were only identified by few participants. These results contrast a study assessing knowledge of CVD in PLWH in the USA whereby >90 % of participants were able to recognize all the established risk factors [36]. In addition, knowledge did not differ by risk status. A potential explanation for our observation is that physicians may not be addressing CVD factors with their patients as our data support. Physicians may regard CVD risk to be very low in PLWH or they may lack awareness of growing threat of CVD in this population. Additionally, the general lack of knowledge may also be the result of underrepresentation of CVD in the health campaigns and biomedical research in SSA [37]. Only >12 years of formal education was independently associated with better knowledge of risk factors, consistent with other survey data [34, 35, 38]. People who have attained higher level of education usually have more health literacy than their counterparts. These results highlight the urgent need of tailored CVD educational programs specifically targeting high-risk PLWH with low education and low literacy skills.

Heart attack symptoms knowledge was much lower than risk factor knowledge. No previous studies have assessed heart attack symptoms knowledge in PLWH. Most participants could not recognize any heart attack symptoms; a finding consistent with previously reported data from other developing countries [39]. In this study, a limited number of the participants identify chest pain as a symptom. This finding contrasts other studies that assessed knowledge in high-risk populations whereby chest pain was the most common identified symptom [34]. Although most of the participants indicated that they would visit a health care facility in the event of a heart attack, not recognizing very basic symptoms such as chest pain may cause major delays in seeking care and worse clinical outcomes [17]. CVD risk status was not related to heart attack symptoms knowledge.

Over two thirds of our participants were found to have at least one CVD yet perception of personal risk for CHD was remarkably low. Our findings are consistent with similar studies in PLWH in the developed countries [33, 36]. In addition, no association was found between CVD risk status and participants’ self perceived risk for the disease. As the health belief model suggested, an individual is likely to take a recommended health action when perceived risk is high. Low perception of CHD risks may inhibit appropriate actions to prevent CHD [16]. It is possible that the general lack of CVD knowledge among participants diminished risk perception [18]. In addition, CVD screening is not a routine part of HIV care in many HIV treatment programs in sub-Saharan Africa therefore prior to this study majority of the participants were not aware of their risk status. As noted in the health belief model, people have a high probability of perceiving themselves at higher risk of disease when the existence of a risk factor is known. Central obesity is also considered a sign of wealth and health by majority of people in Kenya. Therefore, what is considered healthy may not be perceived as such for this population. Another possible explanation for these findings could be the presence of an optimistic, age-related bias. Our study population was made up of relatively young adults and only 16 % were older than 50 years thus they may believe that as individuals they were not at risk for CVD events, which tend to occur most often in an older population. Future studies need to investigate effective ways to communicate CVD risk to PLWH so that these individuals understand their risk of CVD and adopt risk reduction behaviors.

Consistent with overall poor knowledge and low self-perceived risk for heart disease, preventive practices were significantly lacking. Only one out of eleven who indicated that they smoke had attempted to quit smoking. Although over half of our participants were found to be overweight or obese, less than five percent reported actively exercising, attempting to lose weight or modifying their diet to improve their health. Reasons given by participants who reported positive health behaviors were: wanted to feel better and advise form a healthcare professional corresponding to the *perceived benefits* and *cues of acti*on domains of the HBM, respectively.

Knowledge alone is not sufficient to improve cardiovascular health but it is a vital pre-requisite for improving attitude, behaviors and practices towards better cardiovascular health [16]. This is mostly because behavior change is influenced by one’s perception and knowledge that those behaviors could cause harm [18]. Since majority of the participants reported using media as a source of medical information, these could be powerful tool for dissemination information on cardiovascular diseases.

There are several limitations to this study. First, this study enrolled a convenience sample consisting of patients who were actively engaged in HIV care. Findings may differ among those not seeking care. Secondly, men were underrepresented in the study. This is partly related to the fact that in this region women tend to seek care more often than men and are readily available to participate in studies. However, given the dearth of women in the largest studies examining the relationship between HIV and CVD, our findings are relevant to an under-represented population of PLWH [8]. Lastly, the use of an open-ended questionnaire could introduce recall bias resulting in underestimation of knowledge stage of the participants.

## Conclusion

This is the first study to assess risk status, knowledge, perception, and practices of PLWH towards CVD prevention in SSA. The study highlights the high prevalence of CVD risk, lack of knowledge of CVD, and inappropriate attitude towards CHD prevention. Our findings have major implications for HIV healthcare providers in Kenya who will bear the brunt of the projected increased in CVD in PLWH in SSA. Alongside CVD treatment modalities, targeted interventions to improve knowledge and perceptions towards CVD are urgently needed in this population. There is great need to integrate CVD risk assessment into the current HIV infrastructure in SSA.

## Additional file

Additional file 1: Questionnaire. (PDF 75 kb)

## Abbreviations

CVD: Cardiovascular diseases
CHD: Coronary heart diseases
SSA: Sub Saharan Africa
PLWH: People living with HIV
HIV: Human immunodeficiency virus
ART: Antiretroviral therapy
HDL-C: High-density lipoprotein
LDL-C: Low -density lipoprotein
