# Supplementary material for: Cardiovascular health knowledge and preventive practices in people living with HIV in Kenya
Source: BMC Infect Dis. 2015 Oct 14;15:421. doi: 10.1186/s12879-015-1157-8 (PMC4607097; doi:10.1186/s12879-015-1157-8)
Supplement: Additional file 1: — Questionnaire. (PDF 75 kb) [file 12879_2015_1157_MOESM1_ESM.pdf]

HIV CVD KAP Study- Aug 2014

Study ID ______________________________

Todays date ______________________________

Enrollment location
- ☐ AMPATH MTRH clinic
- ☐ Mosoriot clinic
- ☐ Turbo clinic
- ☐ Webuye
- ☐ other

1.Demographics

Gender
- ☐ Female
- ☐ Male
- ☐ Unknown

Date of birth ______________________________

Marital status
☐ Single ☐ Married ☐ Divorced
☐ Widowed ☐ Separated

Occupation ______________________________

II. Medical history-
Any personal or recorded history of?

High Cholesterol	☐ Yes	☐ No	☐ D/K
Diabetes	☐ Yes	☐ No	☐ D/K
High blood pressure	☐ Yes	☐ No	☐ D/K
Stroke	☐ Yes	☐ No	☐ D/K
Myocardial infraction	☐ Yes	☐ No	☐ D/K
Peripheral arterial disease	☐ Yes	☐ No	☐ D/K
Heart failure	☐ Yes	☐ No	☐ D/K

Are you currently prescribed the following?

Lipid lowering drugs	☐ Yes	☐ No	☐ D/K
Blood pressure drugs	☐ Yes	☐ No	☐ D/K
Diabetic drugs	☐ Yes	☐ No	☐ D/K
HIV drugs	☐ Yes	☐ No	☐ D/K

when did you start taking medication? year e.g. 1986 ______________________________

Asprin	☐ Yes	☐ No	☐ D/K

Do you have any history of any of the following in your family?

High Cholesterol	☐ Yes	☐ No	☐ D/K
Diabetes	☐ Yes	☐ No	☐ D/K
High Blood Pressure	☐ Yes	☐ No	☐ D/K
Stroke	☐ Yes	☐ No	☐ D/K
Myocardial infraction (Heart attack)	☐ Yes	☐ No	☐ D/K

III. Social history

A. Tobacco

Do you use tobacco?

☐ Yes ☐ Never ☐ Stopped
☐ Don't' Know/Refuse

If you use tobacco or used to use tobacco, what kind do (did) you usually use (tick all that applies)?

☐ Cigarette
☐ Pipe
☐ Chew
☐ Cigar
☐ Snuff
☐ Other:

B.Alcohol

Do you sometimes drink alcohol?

☐ Yes
☐ No
☐ Stopped

If stopped, how old were you when you stopped? years

____

If you drink alcohol or used to drink alcohol, what kind do (did) you usually drink? (Tick all that apply)

☐ Chang'aa ☐ Liquor
☐ Beer ☐ Wine ☐ Busaa

How often did you have a drink containing alcohol in the last year?

☐ Never
☐ Monthly or less
☐ 2 to 4 times a month
☐ 2 to 3 times per week
☐ 4 to 5 times per week
☐ ‰ä´6 times per week

How many drinks containing alcohol did you have on a typical day when you were drinking alcohol in the past year?

☐ 0 drink
☐ 1 to 2 drinks
☐ 3 to 4 drinks
☐ 5 to 6 drinks
☐ 7 to 9 drinks
☐ 10 or more drinks

How often did you have six or more drinks on one occasion in the past year?

☐ Never
☐ Less than monthly
☐ Monthly
☐ Weekly
☐ Daily or almost daily

C. Physical activity

Does your work involve vigorous-intensity activity that causes large increases in breathing or heart rate? [Such as carrying or lifting heavy loads, digging or construction work, running up hills]	☐ Yes ☐ No
If yes, does it last for at least 10 minutes continuously?	☐ Yes ☐ No
In a typical week, on how many days do you do vigorous-intensity activities? As part of your work? days	____________________
How much time do you usually spend sitting or reclining on a typical day? hrs.	____________________

Nutrition /Diet

In a typical week, on how many days do you eat fruit (like apples, oranges, mangos)? days (one number only)	____________________
On average, how many pieces of fruit do you eat on one of those days? Pieces (one number only)	____________________
In a typical week, on how many days do you eat vegetables (like sukumawiki, managu, cabbage)? Days (one number only)	____________________
On average, how many servings of vegetables do you eat on one of those days? Servings (One number only) One serving = approx. 1 cup full	____________________
How often do you add salt to your food after it is cooked?	☐ Never ☐ Often after tasting<br>☐ Often before tasting

IV. Questions about health in general

In your opinion, what is the greatest health problem for HIV patients today (one answer only, open ended question)?	☐ AIDS ☐ Cancer ☐ Heart Disease<br>☐ Diabetes ☐ Stress<br>☐ Stroke ☐ Psychiatric problems<br>☐ Smoking ☐ Tuberculosis<br>☐ Depression ☐ Stigma<br>☐ Others ☐ Don't know/No answer
What is the leading cause of death in HIV patients? (one answer only, open-ended question) ?	☐ Accidents ☐ Tuberculosis<br>☐ Heart diseases ☐ Cancer<br>☐ Opportunistic infections<br>☐ Diabetes ☐ Stroke<br>☐ Others ☐ Don't know/no answer

V. The next few questions will help us to evaluate your knowledge of cardiovascular diseases (heart diseases) and their risk factors

How do you rate your knowledge on heart disease?
- ☐ Very well informed
- ☐ Well informed
- ☐ Moderately informed
- ☐ Not at all informed
- ☐ Do not know/No answer

Have your ever had a discussion with your doctor/health care worker about heart diseases when talking about your health?
☐ Yes ☐ No ☐ D/K

Are you comfortable talking with your Physician about preventive health options and treatment options regarding your health?
- ☐ Comfortable
- ☐ Somewhat comfortable
- ☐ Not comfortable
- ☐ Do Not Know/No Answer

Within the past year, have you ever seen, heard, or read information about heart disease?
☐ Yes ☐ No

what was your source of information?
- ☐ Magazine/news papers
- ☐ Television ☐ Radio
- ☐ Text message ☐ Family/friends
- ☐ Health care professional
- ☐ Brochure/books ☐ Internet
- ☐ Other sources ☐ Community meetings
- ☐ Don't know

Some people your age get heart disease like hypertension, heart attacks, or heart failure. Do you think you might get one of these conditions? Do you think you are at risk for any of these heart diseases?
☐ Yes ☐ No ☐ D/K

Do you know where to go to be screened for heart diseases?
☐ Yes ☐ No ☐ D/K

Do you know how we screen for heart diseases?
☐ Yes ☐ No ☐ D/K

Have you ever been screened (BP, ECG, ECHO, PE) for heart diseases?
☐ Yes ☐ No ☐ D/K

If answer is No, will you want to be screened for heart diseases?
☐ Yes ☐ No ☐ D/K

I am afraid to be screened for heart diseases because I am fearful of bad news?
☐ Yes ☐ No ☐ D/K

Now I would like you to tell me of all the causes/risks of heart attack about which you know (open ended question)
- ☐ High blood sugar
- ☐ High blood pressure
- ☐ High cholesterol
- ☐ Physical inactivity
- ☐ Drinking alcohol
- ☐ Overweight/obese
- ☐ Stress
- ☐ Smoking/Tobacco use
- ☐ Inherited risk
- ☐ Age
- ☐ Other
- ☐ Do not know answer/No answer

Can you tell me of all the warning signs of heart attack? (open-ended question)

- ☐ Difficulty breathing
- ☐ Dizziness or light headedness
- ☐ Loss of consciousness
- ☐ Back pain
- ☐ Pain in the chest with/without exercise
- ☐ Excessive sweating
- ☐ Vomiting tendency
- ☐ Pain in the teeth or jaw
- ☐ Pain in the abdomen
- ☐ I don't know
- ☐ Others

If someone gets a heart attack at home what will you do or won't do?

Treat at home — ☐ Will do ☐ Won't do ☐ Not Sure

Immediately take the person to the hospital — ☐ Will do ☐ Won't do ☐ Not Sure

Take to a pharmacy/medicine shop — ☐ Will do ☐ Won't do ☐ Not Sure

Wait for appointment with a heart specialist — ☐ Will do ☐ Won't do ☐ Not Sure

Take the patient to a traditional healer — ☐ Will do ☐ Won't do ☐ Not Sure

Give home-made therapy — ☐ Will do ☐ Won't do ☐ Not Sure

Don't know — ☐ Will do ☐ Won't do ☐ Not Sure

VI. Attitude towards cardiovascular risk factor modification

I do want to change my lifestyle activity (e.g. smoking, alcohol) to protect against heart disease. — ☐ strongly agree ☐ somewhat agree ☐ do not know ☐ somewhat disagree ☐ strongly disagree

My present weight is too high for my health — ☐ strongly agree ☐ somewhat agree ☐ do not know ☐ somewhat disagree ☐ strongly disagree

The amount of food I eat is too much, and I would like to cut down — ☐ strongly agree ☐ somewhat agree ☐ do not know ☐ somewhat disagree ☐ strongly disagree

Changing my behavior will reduce my risk of developing heart diseases. — ☐ strongly agree ☐ somewhat agree ☐ do not know ☐ somewhat disagree ☐ strongly disagree

I don't have time to take care of myself — ☐ strongly agree ☐ somewhat agree ☐ do not know ☐ somewhat disagree ☐ strongly disagree

God or a higher power ultimately determines my health, not me.
- ☐ strongly agree ☐ somewhat agree
- ☐ do not know ☐ somewhat disagree
- ☐ strongly disagree

What is your opinion about the following statements

Having Access to more fruits, vegetables and other health foods would greatly lower my risk of developing heart diseases.
- ☐ strongly agree ☐ somewhat agree
- ☐ do not know ☐ somewhat disagree
- ☐ strongly disagree

More awareness programmes for a healthy heart (advertisements, SMS, radio and television shows would help me avoid risk of heart diseases.
- ☐ strongly agree ☐ somewhat agree
- ☐ do not know ☐ somewhat disagree
- ☐ strongly disagree

Health facilities to detect and treat heart diseases are readily available to me.
- ☐ strongly agree ☐ somewhat agree
- ☐ do not know ☐ somewhat disagree
- ☐ strongly disagree

Local volunteers should try to change any adverse health behaviors among people at risk for heart diseases.
- ☐ strongly agree ☐ somewhat agree
- ☐ do not know ☐ somewhat disagree
- ☐ strongly disagree

Smoking bans should be applied to help people avoid heart diseases.
- ☐ strongly agree ☐ somewhat agree
- ☐ do not know ☐ somewhat disagree
- ☐ strongly disagree

VII. This section will tell us more about your current practices towards stronger and healthy heart. In the past year,

Have you had your blood pressure measured? ☐ Yes ☐ No ☐ Not Sure

Have you got a diagnostic test for heart disease? Echo, ECG, Lipid profile)? ☐ Yes ☐ No ☐ Not Sure

Attempted to cut down on unhealthy foods? ☐ Yes ☐ No ☐ Not Sure

Have you tried to quit smoking ☐ Yes ☐ No ☐ Not sure

Have you tried to cut weight/increase physical activity ☐ Yes ☐ No ☐ Not sure

What was the reason for getting the above tests/change your behavior
- ☐ Wanted to feel better
- ☐ I did it for the family
- ☐ Wanting to avoid medications
- ☐ Advised by a health professional/
- ☐ Worried about getting heart diseases because a friend/family member died of it
- ☐ other

VIII. Anthropometric measurements

Systolic blood pressure Right 1 ________________________________

Systolic blood pressure Right 2 ________________________________

Systolic blood pressure Left 3 ________________________________

Systolic blood pressure Left 4 ________________________________

Diastolic blood pressure Right 1 ________________________________

Diastolic blood pressure Right 2 ________________________________

Diastolic blood pressure Left 3 ________________________________

Diastolic blood pressure Left 4 ________________________________

Waist circumference (cm) ________________________________

Hip circumference (cm) ________________________________

8. When were you told you are HIV infected? (Give at least month & year) ________________________________

9. Do you take any medication for your HIV infection?
   - ☐ Yes
   - ☐ No

10. Which HIV medication (ARVs) do you take for your HIV infection? (Tick all appropriate)
    - ☐ Stavudine
    - ☐ Zidovudine
    - ☐ Nevirapine
    - ☐ Lopinavir/ritonavir
    - ☐ Lamivudine
    - ☐ Tenofovir
    - ☐ Efervirenz
    - ☐ Others

11. When did you start taking your HIV medication? (Give at least month & year) ________________________________

12. Have you ever had your HIV medication changed/stopped? ☐ Yes ☐ No

13. When was your HIV medication changed/stopped? (Give at least month & year) ________________________________

14.Which HIV medication (ARVs) were you taking for your HIV infection before?
    - ☐ Stavudine
    - ☐ Zidovudine
    - ☐ Nevirapine
    - ☐ Lopinavir/ritonavir
    - ☐ Lamivudine
    - ☐ Tenofovir
    - ☐ Efervirenz
    - ☐ Others

OPPORTUNISTIC INFECTION (from chart)

WHO Clinical staging ________________________________

Opportunistic infection 1 ________________________________

Opportunistic infection 2 ________________________________

Opportunistic infection 3 ________________________________

Opportunistic infection 4 ________________________________

LABORATORY VALUES (From Chart)

Date of record highest viral load	________________________________
Viral load highest value	________________________________
Viral load lowest value	________________________________
Date of lowest viral load recorded	________________________________
CD4 count highest value	________________________________
date of record highest CD4 value	________________________________
Lowest CD4 value	________________________________
date of record lowest CD4 value	________________________________

LABARATORY MEASUREMENTS

Date of blood draw	________________________________
1.Random blood sugar (mmol/l)	________________________________
2. FBS (mmol/l)	________________________________
3. Total cholesterol (mmol/l)	________________________________
5.LDL - cholesterol (mmol/l)	________________________________
4.Triglyicerides (mmol/l)	________________________________
6.HDL - cholesterol (mmol/l)	________________________________

Body Composition Analyzer

Height (cm)	________________________________
Weight (kilograms)	________________________________
FAT %	________________________________
Fat Mass (Kg)	________________________________
Muscle Mass (Kg)	________________________________
Bone Mass (Kg)	________________________________
Metabolic age (Years)	________________________________
Visceral fat rating	________________________________
BMI	________________________________
Ideal body weight (Kg)	________________________________
Degree of obesity (%)	________________________________
